# Multilevel analysis of factors associated with suicide attempts: Evidence from 2022/2023 Mozambique Demographic and Health Survey

**DOI:** 10.1371/journal.pone.0315648

**Published:** 2025-02-04

**Authors:** Angwach Abrham Asnake, Beminate Lemma Seifu, Bezawit Melak Fente, Hiwot Altaye Asebe, Meklit Melaku Bezie, Yohannes Mekuria Negussie, Zufan Alamrie Asmare, Mamaru Melkam

**Affiliations:** 1 Department of Epidemiology and Biostatistics, School of Public Health, College of Health Sciences and Medicine, Wolaita Sodo University, Wolaita Sodo, Ethiopia; 2 Department of Public Health, College of Medicine and Health Sciences, Samara University, Samara, Ethiopia; 3 Department of General Midwifery, School of Midwifery, College of Medicine & Health Sciences, University of Gondar, Gondar, Ethiopia; 4 Department of Public Health Officer, Institute of Public Health, College of Medicine and Health Sciences, University of Gondar, Gondar, Ethiopia; 5 Department of Medicine, Adama General Hospital and Medical College, Adama, Ethiopia; 6 Department of Ophthalmology, School of Medicine and Health Science, Debre Tabor University, Debre Tabor, Ethiopia; 7 University of Gondar, College of Medicine and Health Science, Department of Psychiatry, University of Gondar, Gondar, Ethiopia; Jawaharlal Institute of Postgraduate Medical Education and Research, INDIA

## Abstract

**Background:**

This issue represents a major global public health concern, accounting for approximately 703,000 deaths each year. Despite Mozambique having the 9th highest suicide rate in the world and the highest in Africa, there is no national data quantifying the burden of suicide attempts. Therefore, this study aimed to assess the prevalence of suicide attempts and identify individual and community-level factors associated with them using mixed-effects logistic regression. This study is crucial for developing early interventions, which can help reduce the risk of suicide and enhance overall mental health outcomes for young people.

**Method:**

The data used in this study were drawn from the 2022/2023 Mozambique Demographic and Health Survey (MDHS). A weighted sample of 10,909 individuals (7,716 males and 3,149 females) aged 15–29 was included. The fitted model was evaluated using AIC and BIC, with the model having the lowest Akaike Information Criterion (AIC) and Bayesian Information Criterion (BIC) is another statistical measure used to assess the quality of a model, similar to the Akaike Information Criterion (AIC). The results of the final model were presented as Adjusted Odds Ratios (AOR) with 95% confidence intervals (CI). Variables were considered statistically significant if their p-value was less than 0.05 in the multivariable analysis.

**Results:**

About 3.60% (95% CI: 3.33%-4.03%) of participants were seriously considered suicide attempt in the past 12 months of before the survey. Educational status, occupation, marital status, depression, anxiety, and geographic region were significant factors associated with suicidal attempt.

**Conclusion:**

The findings of this study enhance our understanding of the intricate relationships between suicide attempts and their predictors. Additionally, the results highlight the need for targeted interventions and mental health promotion strategies that consider the identified individual and community-level factors to reduce suicide rates in Mozambique.

## Introduction

Suicide is defined as the intentional act of self-harm that results in death. Suicidal conduct includes a range of behaviors, such as suicidal ideation (thoughts and ideas), as well as both completed and attempted suicide [1]. A suicide attempt is an intentional effort to take one’s own life that does not result in death [2]. This issue represents a major global public health concern, accounting for approximately 703,000 deaths each year [1]. For every completed suicide, many individuals attempt suicide, and a significant risk factor for suicide in the general population is having previously attempted suicide [3].

Suicide is the 4^th^ most common cause of mortality for people aged 15 to 29 [3]. The prevalence of suicidal attempts was 6.8% in Mexico [4], 7.3% in Tunisia [5], 24% of male attempters and 16% of female in China [6], 3.9% in Haramaya -Ethiopia [7], 8.2% in Addis Ababa-Ethiopia [8], 7.8% in Kenia [9], 17.5% in Ghana [10], and 4% to 18.5% in Mozambique [11, 12]. Male sex, being unemployed, low and middle household income, sexual abuse, frequent conflicts, any psychiatric disorder (i.e. depression, anxiety and bipolar disorders), a family history of suicide, a poor medical condition, stressors/bereavement, and living alone [13–16].

A suicide attempt is a significant risk factor for completed suicides [17]. Evidence indicates that for every adult who dies by suicide, there may be more than 20 others who have attempted it. Evaluating a suicide attempt serves as a warning sign for future suicidal behavior and provides essential information about the individual’s needs and level of distress. Furthermore, the suicide death rate underscores the importance of Target 3.4 of the Sustainable Development Goals, which aims to promote mental health and well-being and reduce premature mortality from noncommunicable diseases by one-third by 2030 [3, 17].

Despite Mozambique having the 9th highest suicide rate in the world [18] and the highest in Africa [19], there are no national studies quantifying the burden of suicide attempts. Therefore, this study aimed to assess the prevalence of suicide attempts and identify individual and community-level factors associated with them using mixed-effects logistic regression.

## Method

### Study design, setting, and population

This study utilized a cross-sectional design. The data were obtained from the 2022/2023 Mozambique Demographic and Health Survey (MDHS). While the MDHS provides estimates for the nation’s capital, Maputo, it was designed to cover all ten provinces—Niassa, Cabo Delgado, Nampula, Zambézia, Tete, Manica, Sofala, Inhambane, Gaza, and Maputo—as well as both urban and rural areas. A total of 619 enumeration areas were selected using a probability distribution that was proportionate to size, determined by explicitly counting the number of households within each stratum [20]. A weighted sample of 10,909 individuals (7,716 males and 3,149 females) aged 15–29 was included in this study. This age group was the focus because they face unique challenges that make them particularly vulnerable to suicide attempts. Understanding these challenges is essential for developing early interventions aimed at reducing suicide risk and improving overall mental health outcomes for young people.

### Dependent variables and definitions

Suicidal attempts were the dependent variable of this study. It was measured by a question “Have you tried to attempt suicide seriously in the past 12 months?” in MDHS.

### Independent variables and definitions

Both individual and community-level variables were included in this study. The age, educational attainment, wealth index, marital status, occupation, and history of mental illness of the respondents were individual-level predictors. Moreover, depression and anxiety were also considered as predictors. A score more than the cutoff of 10, was considered as participants had anxiety and depression [21, 22]. The wealth index in the MDHS was generated using principal component analysis. The survey classified all interviewed households into five wealth quintiles: poorest, poorer, middle, richer, and richest. For this study, these were grouped into two categories: poor (comprising the poorest and poorer quintiles) and non-poor (including middle, richer, and richest quintiles). Geographical location and place of residence were community-level factors.

### Statistical analysis and model building

STATA version 18 was used for data analysis. Tables were used to provide descriptive statistics. To account for the nonproportional distribution of the sample between urban and rural regions and potential variations in response rates, a sampling weight was applied in each analysis. Proper representativeness of the study was thus ensured both nationally and regionally. A multilevel binary logistic regression model was used to determine the effects of each independent variable on suicidal attempts while accounting for the clustering effects of the DHS data. In datasets with a nested structure, like the MDHS, individuals within the same cluster (such as households or communities) often share common characteristics, leading to some correlation between observations. When this correlation is overlooked and only individual-level factors are analyzed, it violates the assumption that observations are independent. In contrast, analyzing both group-level and individual-level variations together has several benefits. It allows for a better understanding of how community and individual factors impact health outcomes. By accounting for the clustering in the data, this approach leads to more efficient estimates of regression coefficients. To capture both fixed effects at the individual and community levels, along with random effects for variation between clusters, a two-level mixed-effects logistic regression analysis was used in this study [23]. To capture the mixed effects, including fixed effects for individual and community-level factors and a random effect for variation between clusters, a two-level mixed-effects logistic regression analysis was applied in this study. The clustered structure of the data, along with the variations within and between communities, was addressed by assuming that each community has a unique intercept (β₀) and a fixed coefficient (β). The level of community variation was quantified using the Intra-class Correlation Coefficient (ICC), which was calculated as follows:

$$\text{ICC}=\frac{s_{b}^{2}}{s_{b}^{2}+s_{w}^{2}}$$

Where $s_{b}^{2}$ = variance between clusters, $s_{w}^{2}$ = variance within the cluster [24].

We computed the ICC, Median Odds Ratio (MOR), and Likelihood Ratio (LR) test to assess the heterogeneity among clusters. When we randomly choose two people from two clusters during data collection—one with a high risk of suicide attempts and the other with a low risk—the MOR calculates the variation in suicidal attempts.


$$\text{MOR}=\exp\sqrt{\left(2*\partial^{2}*0.6745\right)}∼\text{MOR}=\exp\;(0.95*\partial )$$

*∂*^2^ indicates that cluster variance [25].

Variables with a p-value less than 0.20 in bivariable analysis and those found important in the literature were considered for multivariable multilevel binary logistic regression analysis. For the multivariable multilevel binary logistic regression, four models were built. In the first model, there were no explanatory factors; in the second, individual-level variables were fitted; in the third, community-level variables were fitted; and in the fourth, both individual- and community-level variables were fitted simultaneously. The fitted model was compared by using deviance and the lowest deviance was considered as the best-fit model. Finally, the good-fitted model’s Adjusted Odds Ratio (AOR) and 95% confidence interval (CI) were disclosed., and variables were considered statistically significant if it’s p-value < 0.05 in the multivariable analysis.

### Ethical consideration

No participant consent or ethical approval was required for this study because it was a secondary data analysis of survey data that the DHS program (https://data.dhsprogram.com/) made publicly available. The survey protocol, which comprised the questions for all DHSs, was examined and authorized by the Ethical Review Board of International Consulting and Fulfillment (ICF’s Inc.). We requested authorization from MEASUREDHS and ICF to access and use their data, and they complied. The data file contained neither personal names nor residential addresses.

## Results

### Characteristics of the participants

A weighted sample of 10, 909 aged 15–29 years old participants were included in this study. The majority (71.19%) of the participants were female and 60.57% of participants resided in rural areas. About 1739 (15.94%) of participants had no education. The majority (57.90%) of them had not worked and 18% of them working on agriculture. More than half (50.92%) of the participants were married/living together. About 706 (6.47%) and 725 (6.46%) of participants had depression and anxiety respectively (**Table 1**).

**Table 1:** Characteristics of participants (weighted sample = 10,909).

Variables	Frequency	Percent (%)
**Age (in years)**		
15–19	4353	39.90
20–24	3613	33.12
25–29	2943	26.98
**Sex**		
Female	7766	71.19
Male	3143	28.81
**Educational status**		
No education	1738	15.94
Primary	4829	44.26
Secondary and higher	4342	39.80
**Occupation**		
Agricultural/self-employed	1954	18.00
Not working	6283	57.90
Professional/technical	216	1.99
Clerical	50	0.46
Sales	1048	9.66
Services	174	1.60
Skilled manual	628	5.79
Unskilled manual	500	4.61
**Wealth status**		
Poor	3999	36.66
Non-poor	6910	63.34
**Marital status**		
Married/living together	5555	50.92
Single	4559	41.79
Widowed/divorced/separated	794	7.28
**Suicide attempt**		
No	10,409	96.36
Yes	393	3.64
**Depression**		
No	10203	93.53
Yes	706	6.47
**Anxiety**		
No	10185	93.36
Yes	725	6.46
**Residence**		
Urban	4302	39.43
Rural	6607	60.57
**Region**		
Maputo	983	9.01
Niassa	740	6.79
Cabo delgado	570	5.22
Nampula	2634	24.14
Zambezia	1936	17.75
Tete	1067	9.78
Manica	757	6.94
Sofala	805	7.37
Inhambane	386	3.54
Gaza	500	4.59
Cidade de Maputo	531	4.87

### Prevalence of suicide attempt

**Fig 1:**
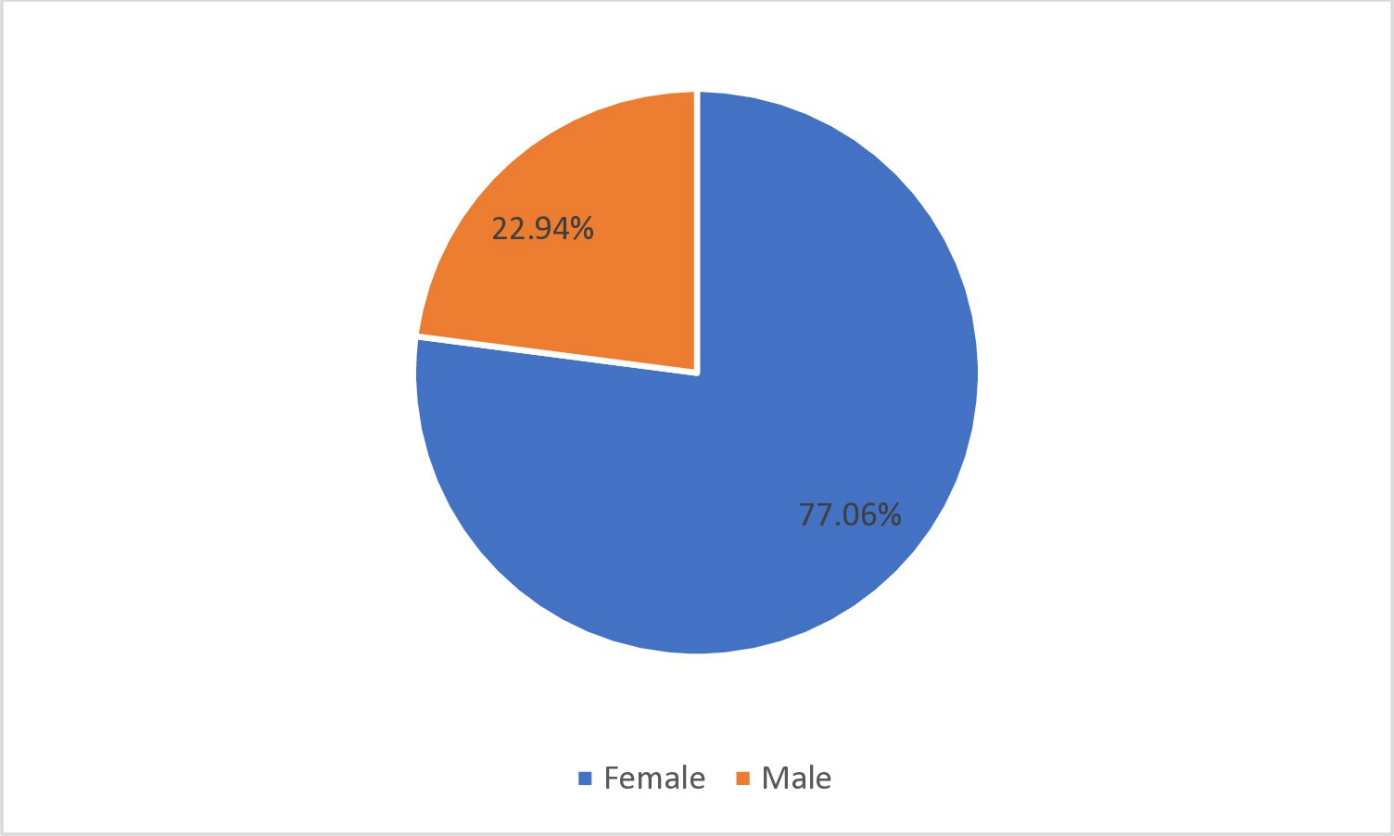
The distribution of suicidal attempt across gender.

About 3.6%, (95% CI: 3.33%-4.03%) of participants were seriously considered suicide attempt in the past 12 months of before the survey (Fig 1).

### Random effect and model compression

Since the LR test was statistically significant (p = 0.0001), the odds of attempting suicide differed statistically considerably between clusters in the null model. Moreover, the MOR showed that participants would have a 1.68 times higher chance of attempting suicide if two individuals were randomly selected from separate clusters and transferred from the cluster with a lower probability to the cluster with a higher probability. Consequently, a multilevel binary logistic regression model was fitted, with the lowest deviance value being used to choose the final model (Table 2).

**Table 2:** Random effect and model comparison.

Random effect and model comparison	Null Model	Model I (individual-level variables only)	Model II (community-level variables only)	Model III (both individual and community level variables)
LR test	<0.001	<0.001	<0.001	<0.001
LL	-1855	-1771	-1835	-1771
AIC	3361	3298	3332	3297
BIC	3376	3436	3426	3416
ICC	8.3%	6.1%	6.0%	5.1%
Deviance	3714	3583	3696	3581
MOR	1.68	1.55	1.63	1.63

### Factors associated with suicidal attempts

Educational status, occupation, marital status, depression, anxiety, and geographic region were significant factors associated with suicide attempts in the multivariable analysis.

The odds of attempting suicide were reduced by 27% (AOR = 0.73, 95% CI: 0.53, 0.98) and 37% (AOR = 0.63, 95% CI: 0.44, 0.90) among participants with primary and secondary/higher education, respectively, compared to those with no education. In comparison to individuals working in agriculture or who are self-employed, professional and technical workers had 1.97 times higher odds of attempting suicide (AOR = 1.97, 95% CI: 1.05, 3.68). Additionally, widowed, divorced, or separated individuals had 1.51 times higher odds of suicide attempts (AOR = 1.51, 95% CI: 1.08, 2.10) compared to married participants. Individuals with depression and anxiety had 3.92 times (AOR = 3.92, 95% CI: 2.69, 5.71) and 1.50 times (AOR = 1.50, 95% CI: 1.01, 2.26) increased odds of suicide attempts, respectively. Furthermore, compared to participants residing in Maputo, those from Niassa, Nampula, and Inhambane had a 54% reduction in the odds of suicide attempts (AOR = 0.46, 95% CI: 0.28, 0.82), 40% reduction (AOR = 0.60, 95% CI: 0.37, 0.99), and 53% reduction (AOR = 0.47, 95% CI: 0.25, 0.88), respectively (Table 3).

**Table 3:** Factors associated with suicide attempt in Mozambique.

Variables	COR with 95% CI	AOR with 95% CI
**Age (in year)**		
15–19	1	1
20–24	1.22 (0.96, 1.55)	1.13 (0.88, 1.46)
25–29	1.11 (0.85, 1.44)	0.94 (0.69, 1.27)
**Sex**		
Female	1	1
Male	0.78 (062, 0.99)*	0.82 (0.62, 1.08)
**Educational status**		
No education	1	1
Primary	0.93 (0.67, 1.31)	0.73 (0.53, 0.98)*
Secondary and higher	1.09 (0.78, 1.52)	0.63 (0.44, 0.90)*
**Employment**		
Agricultural/self-employed	1	1
Not working	1.34 (0.97, 1.86)	1.32 (0.93, 1.88)
Professional/technical	2.47 (1.38, 4.43)*	1.97 (1.05, 3.68)*
Clerical	1.69 (0.50, 5.67)	1.38 (0.40, 4.71)
Sales	1.69 (1.06, 2.41)*	1.27 (0.82, 1.97)
Services	1.86 (0.96, 3.62)	1.53 (0.76,3.08)
Skilled manual	1.54 (0.95, 2.48)	1.56 (0.93, 2.62)
Unskilled manual	1.53 (0.93, 2.54)	1.46 (0.87, 2.45)
**Wealth index**		
Poor	1	1
Non-poor	1.54 (1.18, 2.01)*	1.34 (0.99, 1.81)
**Marital status**		
Married/living together	1	1
Single	1.04 (0.84, 1.30)	0.98 (0.75, 1.28)
Widowed/divorced/separated	1.79 (1.27, 2.53)*	1.51 (1.08, 2.10)*
**Depression**		
No	1	1
Yes	3.23 (2.29, 4.54)*	3.92 (2.69, 5.71)*
**Anxiety**		
No	1	1
Yes	2.04 (1.39, 2.99)*	1.50 (1.01, 2.26)*
**Place of residence**		
Urban	1	1
Rural	0.72 (0.57, 0.91)*	0.79 (0.61, 1.04)
**Geographic region**		
Maputo	1	1
Niassa	0.40 (0.23, 0.71)*	0.46 (0.28, 0.82)*
Cabo Delgado	0.63 (0.38, 1.04)	0.94 (0.58, 1.51)
Nampula	0.35 (0.20, 0.61)*	0.60 (0.37, 0.99)*
Zambezia	0.95 (0.59, 1.54)	1.19 (0.73, 1.93)
Tete	0.84 (0.52, 1.36)	1.05 (0.64, 1.70)
Manica	0.66 (0.40, 1.01)	0.74 (0.45, 1.22)
Sofala	0.95 (0.60, 1.51)	1.07 (0.67, 1.69)
Inhambane	0.43 (0.23, 0.80)*	0.47 (0.25, 0.88)*
Gaza	0.53 (0.31, 0.91)*	0.63 (0.37, 1.07)
Cidade de Maputo	1.06 (0.67, 1.69)	0.98 (0.62, 1.56)

*indicates p-value <0.05

## Discussion

The aim of this study was to assess the prevalence of suicide attempts and identify individual and community-level factors associated with suicide attempts using mixed-effects logistic regression. Approximately 3.60% of participants (95% CI: 3.33%-4.03%) reported seriously considering a suicide attempt. Significant factors associated with suicide attempts included educational status, occupation, marital status, depression, and geographic region.

About 3.6%, (95% CI: 3.33%-4.03%) of participants were seriously considered suicide attempt in this study. In line with a study from Haramaya -Ethiopia [7]. Lower than previous studies in Mozambique [12], Ghana [10], Kenia [9], Ethiopia [8], Mexico [4], Tunisia [5], and China [6]. This suggests that the factors influencing suicidal behavior in different contexts are complex. These disparities may be due to population differences, especially since a prior study in Mozambique focused on school-aged adolescents, while this study included individuals aged 15–29. Adolescence is a period of significant physical and psychological transition, which can trigger anxiety, stress, and a sense of losing control, potentially leading to suicidal thoughts and attempts [26]. Additionally, some characteristics of the population in this study did not include hospitalized patients, so this discrepancy could be related to both sample size and socioeconomic differences.

The odds of attempting suicide were lower among participants with primary and secondary/higher education compared to those with no education. In line with the studies in India [27], Iran [28], and United Stats [29, 30]. The possible reason might be those who have low education may have a lack of insight into or awareness of psychiatric conditions that could be associated with increased suicide risk. In comparison to those working in agriculture/self-employed, professional/technical workers had increased odds of suicidal attempt. This finding convergent with study from United States [31]. Adults who work in a given occupation may share similar work-related stresses and working in agriculture/self-employed may reduce work related stress [31]. Education can improve awareness of mental health issues, but those in high-stress professional roles might have less time or fewer opportunities to seek help due to demanding schedules. Moreover, higher education often equips individuals with better problem-solving skills and coping strategies, whereas those in high-stress careers might struggle with these due to the nature of their work [32, 33].

Widowed/divorced/separated individuals had increased suicidal attempts compared to married participants. Convergent with studies from Swedin [34] and China [35]. This may be the result of decreased household/wife income, a higher chance of poverty, and a higher possibility of single parenting after divorce or widowhood [36].

Individuals with depression and anxiety had increased odds of suicidal attempts. This finding is supported by studies from China [37], and Australia [38]. This could be due to risks sharing of suicide attempt with depression and anxiety [39]. Compared to participants residing in Maputo, those from Niassa, Nampula, and Inhambane showed a reduced likelihood of suicidal attempts. This may be due to in these more traditional areas, cultural norms that emphasize community cohesion may discourage suicidal behavior, as community welfare is often prioritized over individual distress, providing a protective buffer against mental health crises [40]. Moreover, fewer urban stressors in these regions, such as lower levels of overcrowding, crime, and living costs, which are often linked to mental health challenges in larger cities like Maputo [41].

### The strengths and weakness of this study

The results of this study are nationally representative since it used a large sample size and national data. The use of a multilevel statistical approach enables to account the hierarchical character of the data. Since this study used self-reported measures of suicidal attempts, it may lead to recall bias and social desirability bias. This study does not prove causation, because of its cross-sectional design, which just demonstrates the relationships between suicide attempt and individual and community-level factors.

## Conclusion

The results of this study enhance our understanding of the complex relationships between suicide attempts and their predictors. Furthermore, these findings underscore the necessity for targeted interventions and mental health promotion that consider the identified individual and community-level variables to reduce suicide rates in Mozambique.
